# CHARACTERIZATION OF DYNAMIC ADAPTATION TO STRESSORS USING MULTISYSTEM STIMULUS–RESPONSE DATA

**DOI:** 10.1093/geroni/igae098.0203

**Published:** 2024-12-31

**Authors:** Karen Bandeen-Roche, Jiafeng Zhu, Qian-Li Xue, Brian Buta, Thomas Laskow, Jeremy Walston, Ravi Varadhan

**Affiliations:** Johns Hopkins University, Baltimore, Maryland, United States; Northwestern University, Evanston, Illinois, United States; Johns Hopkins University, Baltimore, Maryland, United States; Johns Hopkins University, Baltimore, Maryland, United States; Johns Hopkins University, Baltimore, Maryland, United States; Johns Hopkins University, Baltimore, Maryland, United States; Johns Hopkins University, Baltimore, Maryland, United States

## Abstract

Resilience has emerged as a major gerontological concept aiming to promote more consistently positive outcomes following stressors. Achieving this aim relies on determining mechanisms underlying capacity to respond resiliently. This paper addresses the hypothesis that physical aspects of said capacity arise considerably from the fitness of one’s physiology governing stress response, conceptualized as a dynamical system. We leverage the Study of Physical Resilience and Aging (“SPRING”) pilot and Phase II studies, which implemented stimulus-response experiments to characterize physiological fitness in older adults scheduled for major stressors of either total knee replacement, incident hemodialysis, or bone marrow transplant for hematological cancers. We develop physiologic resilience capacity measures using time series from Holter monitor assessment, cortisol response to adrenocorticotropic hormone stimulation, and repeated diurnal salivary cortisol assessment. Principal components (PCs) of the multivariable summaries from these tests were derived using SPRING pilot data. The first two components represented “steady state” and “adaptation” mechanisms. Steady state and adaptation PC scores were separately regressed on major stressor type, hypothesized static physiologic indicators (physical frailty phenotype, self-report of health), and personal characteristics. Substantial association was manifest by stressor type and the static physiologic indicators: for both PCs, average scores were 0.6 SD higher for persons deemed robust versus prefrail/frail after stressor type adjustment (p< 0.05). Cross-validation analyses using Phase II data, as well as analyses to discriminate resilience phenotypes and outcomes post-stressor with these data, will be reported. Our study lays groundwork to better forecast and foster resilience of older adults to clinical stressors.

